# A General Analytic Approach for Rapid Diagnostics by a Simple Algorithm for Fluorescence Single Molecule Counting

**DOI:** 10.3390/bios16050270

**Published:** 2026-05-08

**Authors:** Juiena Hasan, Sangho Bok

**Affiliations:** Department of Electrical and Computer Engineering, University of Denver, Denver, CO 80208, USA; juiena.hasan@du.edu

**Keywords:** single molecule detection, fluorescence microscopy, blinking analysis, threshold-based algorithm, digital molecular counting, plasmonic biosensing, rapid diagnostics

## Abstract

Accurate biomolecule quantification at ultralow concentrations remains a major challenge because conventional ensemble assays report population averaged signals and therefore lose sensitivity in low-abundance regimes. Single molecule fluorescence counting can overcome this limitation by converting emission into discrete digital events, but practical implementation is often hindered by manual inspection, limited reproducibility, and the complexity of machine learning based analysis. Here, we present a simple and general analytical framework for rapid single molecule detection based on a deterministic threshold algorithm that exploits the temporal signature of fluorescence blinking. The method operates directly on time resolved fluorescence image stacks, applies median filter-based noise suppression, and identifies candidate single molecule events from consecutive frame-to-frame intensity transitions without the need for training data or model fitting. Applied to Alexa Fluor 488, Alexa Fluor 647, and Rhodamine Red–X datasets, the approach reproduced the concentration dependent trends observed by manual counting, while providing more standardized detection under weak signal and high background conditions. Dye specific operating thresholds yielded robust counting behavior and preserved approximately linear concentration dependent response across the tested range. Compared with manual analysis, which required inspection of only selected grid regions, the automated workflow processed full movie stacks and reduced analysis time from ~3 h to ~20 min per concentration, corresponding to an approximately 9-fold gain in efficiency. These results establish an interpretable, computationally lightweight, and experimentally adaptable strategy for fluorescence single molecule counting that can support rapid diagnostics and provide a practical foundation for future extensions in automated localization, clustering, and real time molecular analysis.

## 1. Introduction

Accurate quantification of biomolecules is central to advances in molecular biology, clinical diagnostics, and translational medicine. Conventional ensemble assays such as gel.

Electrophoresis [1], enzyme-linked immunosorbent assays [2], and colorimetric detection methods [3] have enabled broad analytical capabilities, yet they fundamentally report population averaged signals. As a consequence, their sensitivity is constrained when targets are present at extremely low abundance, particularly in early disease states where biomarker concentrations may fall below picomolar levels [4]. Signal amplification strategies can partially address this limitation, but they often introduce additional complexity, extended processing time, and potential sources of variability [5]. These constraints motivate the development of analytical methodologies capable of resolving and enumerating individual molecular entities with high specificity and minimal sample consumption.

Single molecule (SM) counting provides a digital framework for molecular quantification that overcomes many of these limitations [6,7]. In contrast to ensemble intensity measurements, SM approaches resolve discrete fluorescence events arising from individual molecular entities, that is, single labeled target molecules detected one at a time, while minimizing interference from background noise [8]. When each molecular entity is associated with a detectable fluorescent signal, counting individual events yields absolute molecular numbers without reliance on bulk calibration curves [9]. This transition from analog intensity readouts to digital enumeration enhances analytical robustness, improves sensitivity, and reduces susceptibility to nonlinear amplification artifacts [4,5]. Consequently, SM counting enables detection in regimes where conventional assays fail, including femtomolar and subfemtomolar concentration ranges [10].

Beyond ultrasensitive detection, SM counting offers mechanistic insight into molecular organization and dynamics [11]. Direct enumeration enables determination of intracellular protein abundance, stoichiometry within macromolecular complexes, and heterogeneity across nanoscale assemblies. Such quantitative resolution is essential for understanding receptor oligomerization [12], enzyme turnover [13], viral packaging [14], and nanoparticle functionalization [15]. By resolving individual molecular contributions rather than averaged ensemble responses, SM counting reveals static and dynamic heterogeneity that remains inaccessible to bulk measurements [5].

Multiple experimental strategies have been developed to implement SM counting. Confocal microscopy defines a femtoliter scale detection volume through spatial filtering, enabling enumeration of fluorescent bursts as labeled molecules transit the focal region [16,17]. Total internal reflection fluorescence microscopy restricts excitation to an evanescent field near an interface, thereby minimizing background from the bulk solution and enhancing signal to noise ratios for surface bound species [18]. Photobleaching step analysis enables stoichiometric determination by counting quantized intensity decreases within diffraction limited spots as individual fluorophores bleach [14,19]. Super resolution localization approaches extend this paradigm by resolving stochastically activated fluorophores with nanometer precision, enabling spatially resolved counting in dense molecular environments [20,21]. Each of these techniques transform fluorescence signals into discrete countable events, thereby implementing a digital analytical principle. Among these approaches, fluorescence blinking provides a particularly informative temporal signature for SM identification [5,22]. At the SM level, stochastic transitions between radiative and non-radiative states arise from photophysical or redox processes, generating characteristic ON and OFF intensity fluctuations [23]. These discrete temporal transitions distinguish true single emitters from slowly varying background signals and enable robust identification without requiring spatial separation beyond the diffraction limit. By exploiting the temporal structure of fluorescence fluctuations, blinking based strategies facilitate direct molecular enumeration while preserving compatibility with standard optical configurations.

Fluorescence blinking, observed since the earliest demonstrations of SM fluorescence, is often cited as definitive proof of SM detection [24,25]. However, it is generally considered detrimental, as intermittent OFF—states, arising from reactive transient species such as triplet excited states and radical ion states [26,27], disrupt FRET efficiency [28], hinder signal tracking, and accelerate photobleaching. Consequently, most studies employ stabilizing reagents to suppress blinking, with strategies such as the reducing and oxidizing system (ROXS) approach [29,30] developed by Tinnefeld and coworkers to quench these reactive intermediates. Under suitably controlled conditions, however, blinking can be repurposed as an informative signature for detection. In parallel, recent machine learning based approaches have shown that SM fluorescence traces can be automatically classified and screened with improved sensitivity and specificity, highlighting the growing role of data driven algorithms in extracting weak but meaningful signals from complex blinking behavior [31,32,33]. To address the low signal to noise ratio associated with blinking, Woods et al. developed a cost effective plasmonic grating platform that enables detection and analysis of chemical and biological analytes at the SM level using a standard upright epi fluorescence microscope [34]. Plasmonic gratings exploit the optical response of noble metals to couple incident light to surface plasmon modes at the metal dielectric interface through surface plasmon resonance (SPR) [35,36]. When the metal is patterned into a periodic nanoscale grating, phase matched plasmon excitation can be achieved under visible illumination and near normal incidence, eliminating the need for prism-based coupling geometries. This plasmonic excitation produces a strongly enhanced evanescent field that increases fluorophore excitation near the surface and promotes surface plasmon coupled emission (SPCE) [37]. Such enhancement is critical because fluorescence from individual molecules is ordinarily too weak to be distinguished from background with sufficient contrast in a conventional epi fluorescence microscope, whereas the plasmonic grating amplifies the signal sufficiently to enable reliable SM counting on this platform [38].

SM fluorescence microscopy enables direct observation of stochastic molecular emission events, which supports quantitative analyses of emitter abundance and dynamics but so far been limited by the expensive optics. However, reliable single molecule background (SM–BG) separation is frequently performed through manual inspection of frames or manual selection of representative signal and background locations. This manual procedure is time consuming and labor intensive because SM imaging experiments generate large spatiotemporal datasets consisting of high resolution image frames (e.g., 1920 × 1440 pixels) acquired across extended time sequences, is difficult to reproduce across analysts, and is sensitive to subjective decision making, particularly when signal to noise ratio is low and blinking events are sparse [24].

Machine learning (ML), a subset of artificial intelligence, enables computer systems to learn from data and make predictions without explicit programming [39,40]. By leveraging algorithms that identify patterns in large datasets, ML offers the advantage of handling vast amounts of information in relatively short periods, making it a powerful tool in medical imaging, biomedical diagnostics, and drug discovery [41]. ML has been applied to model stochastic molecular dynamics and to distinguish signals from background noise with accuracy comparable to manual counting [31]. Wu and Rifkin have extended a spot-centric pipeline that identify, localize, and classify biologically meaningful intensity maxima in challenging datasets, such as wide field epifluorescence smFISH images of nematode embryos with high background fluorescence [42]. The study here exemplifies a supervised ML approach to SM counting in which candidate fluorescence intensity maxima are characterized by engineered features and classified as true molecular events or background using a random forest model. The total molecular abundance is subsequently obtained by enumerating the validated spots, with probabilistic calibration enabling estimation of counting uncertainty. Such frameworks recast SM detection as a data driven classification task followed by digital event aggregation. Extending this paradigm to multiplexed molecular profiling, the work in [43] presents an ML enabled SM counting pipeline for microRNA analysis. Individual surface immobilized microRNA reporter complexes are detected as diffraction limited spectral point spread functions and classified into target identities using a supervised model. Spectral barcodes encoded by fluorophore pairs are converted into countable SM events through automated spot detection, principal component analysis-based feature compression, and support vector machine classification. The final microRNA abundance profile is reconstructed by enumerating classified events per target, with confusion matrix-based calibration applied to correct mixture ratios and quantify counting uncertainty. Another ML based strategy for SM counting has been introduced through deep neural network analysis of photobleaching time traces [44,45]. In this framework, a convolutional and LSTM hybrid architecture directly identifies discrete photobleaching steps from raw fluorescence trajectories, enabling automated inference of molecular stoichiometry. Molecular counts are subsequently obtained by enumerating model predicted bleaching transitions while suppressing interference from stochastic blinking events.

Although ML approaches have demonstrated strong performance in fluorescence image analysis, their application to SM detection introduces practical and methodological constraints that limit their suitability in resource constrained or highly variable experimental settings [46]. Supervised models require extensive, accurately labeled training datasets [32], however, as noted previously, the annotation of stochastic blinking events under low signal to noise conditions poses substantial challenges for achieving consistent standardization across experimental environments and laboratories. Model performance is further influenced by sensitivity to domain shift, since variations in optical configuration, excitation intensity, detector characteristics, and plasmonic substrate geometry can alter signal statistics and degrade generalization. In addition, training and deploying ML models on high resolution spatiotemporal datasets impose nontrivial computational and memory demands, while complex learned parameters reduce methodological transparency and complicate precise reporting of analytical criteria [47].

Given the disadvantages of manual annotation, automation is essential for reducing the burden, enforcing reproducibility through explicitly defined rules, and enabling scalable analysis across larger fields of view and extended acquisition periods. In this context, automated detection refers to algorithmic identification of SMs like intensity transients with minimal human intervention. SM emission events exhibit a characteristic temporal signature defined by rapid intensity transitions across consecutive frames that are inconsistent with slowly varying background fluctuations. The presented work formalizes this distinction as a deterministic decision problem over spatiotemporal fluorescence measurements and introduces an interpretable threshold-based detection pipeline that isolates candidate SM events using explicitly defined consecutive frame intensity differences. The proposed framework is designed as a lightweight and transparent screening tool that can be reported precisely and reapplied consistently across datasets without dependence on training data or model retraining. Unlike supervised ML pipelines, the proposed framework identifies candidate SM events without requiring labeled training data, parameter tuning, or model fitting. The method instead relies on explicitly defined temporal intensity-difference criteria, which makes the detection logic transparent and directly reproducible. This provides a practical advantage in resource-constrained settings and under variable experimental conditions where training data standardization and model generalization may be difficult to maintain. Its computational simplicity enables efficient processing of large image stacks while preserving analytical clarity. By grounding detection in physically motivated signal characteristics rather than learned representations, the method establishes a reproducible and analytically tractable foundation for subsequent extensions, including spatial clustering of detections, event localization, and automated molecule counting.

## 2. Materials and Methods

### 2.1. Immunoassay by Using Plasmonic Grating

Plasmonic gratings were fabricated using a soft lithography approach following previously reported methods [34,48]. In brief, an elastic mold was produced by curing Dow Sylgard^®^ 184 polydimethylsiloxane (PDMS, Ellsworth Adhesives, Germantown, WI, USA) mixed at a 5:1 ratio over a cleaned, halved HD-DVD for 24 h at 50 °C and 55% relative humidity. Glass microscope slides (Fisherbrand, Fisher Scientific, Hampton, NH, USA) were first washed with a dilute soap solution and deionized water, then dried under a stream of nitrogen. The slides were subsequently cleaned in a 3:1 H_2_SO_4_:H_2_O_2_ (Piranha solution) for 15 min, dip-rinsed twice in deionized water, washed thoroughly under a continuous flow of deionized water, and dried again under flowing nitrogen. The cured PDMS replicated from the HD-DVD was cut into square slabs, spin-coated with 3% *w*/*w* GR650F polymethylsilsequioxane (PMSSQ, Techneglas Inc., Perrysburg, OH, USA) in ethanol, and then stamped onto the cleaned glass slides. Owing to differences in surface energy, the mold was carefully peeled away, leaving the grating pattern transferred onto the glass surface. The patterned slides were then exposed to vapor treatment with 1:1 3-aminopropyltriethoxysilane (APTES) in ethanol, pre-baked at 60 °C for 3 h, and baked at 400 °C for 1 h. This was followed by deposition of a 100 nm silver layer using an AJA RF magnetron sputtering system. Immediately afterward, the silver gratings were transferred to an atomic deposition system, where a 10 nm alumina coating was applied to protect the silver from degradation.

**Figure 1:**
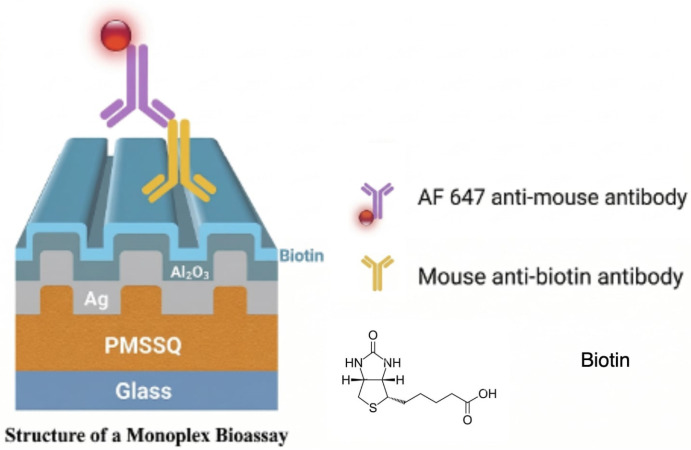
The schematic of monoplex bioassay with AF 647. The monoplex bioassays have been repeated for AF 488 labeled antibody and RRX labeled antibody.

For monoplex detection on the plasmonic gratings, the immunofluorescence assay was designed using the established principles of enzyme-linked immunosorbent assay (ELISA). Primary antibodies were sourced from Invitrogen (Carlsbad, CA, USA), while secondary antibodies were obtained from Jackson Immunoresearch Laboratories Inc. (West Grove, PA, USA). Biotin molecules were immobilized on the grating surface through the activation of the carboxyl group on biotins, facilitating their attachment to an amine-reactive ester on the grating surface following APTES treatment. To achieve biotin binding, an activation buffer (0.1 M MES (2–[morpholino] ethanesulfonic acid), 0.5 M NaCl, pH 6.0) was prepared with 4 mg EDC and 11 mg sulfo-NHS, to which 5 µg of biotin was added. The mixture was incubated for 15 min at room temperature. The activated biotin solution was then applied to the APTES-treated gratings, and the pH was adjusted to ~7 with 100 µL of PBS (pH 8). The biotin–APTES mixture was incubated for 2 h at room temperature under gentle agitation. Following incubation, the gratings were washed three times with PBS. Blocking was performed using 5% BSA in PBS for 1 h, followed by three PBS washes. The blocked gratings were then used for fluorescence assays. Anti-biotin antibodies, with concentrations ranging from 1 fg/mL to 100 pg/mL, were applied to the gratings and incubated for 1 h. After washing the gratings three times with PBS, a signal antibody conjugated with Rhodamine Red–X (RRX) at 5 µg/mL was applied and incubated for 1 h. Finally, the gratings were washed three times, and fluorescence measurements were performed. There were two additional monoplex bioassays with signal antibodies with Alexa Fluor 488 (AF 488) and Alexa Fluor 647 (AF 647), respectively, as shown in Figure 1.

For the monoplex bioassays, large area (1 in × 1 in) coverslips were placed over the slides. Samples were then imaged using an ORCAFlash 2.8 CMOS camera at 1 s integration time on an epi fluorescence microscope (BX51WI from Olympus, Needham, MA, USA) using a 60× water-immersion objective. At least sixty consecutive images were obtained, and the acquired images were analyzed by using ImageJ software (version 1.53) to quantify the fluorophore labeled antibodies. The images exhibited blinking fluorescence indicating SMs were counted.

### 2.2. Manual Counting of SMs

Manual identification of SM blinking events was performed by a trained observer through frame-by-frame inspection of the time resolved sequence. A blinking event was operationally defined as a localized fluorescence transient exhibiting a rapid intensity increase followed by a decrease across consecutive frames, distinguishable from slowly varying background fluctuations or static features. To enable systematic spatial quantification, a grid composed of square regions of 36 μm^2^ area was overlaid onto each image stack. Each grid region was examined sequentially across the temporal axis, and pixel regions demonstrating characteristic blinking behavior were marked and counted as SM events. Persistent background fluorescence, gradual intensity drifts, and non-transient features were excluded based on their temporal profiles. For each fluorophore concentration, event counts within each grid square were normalized by the corresponding area to obtain SM density expressed as counts per micrometer squared. Measurements were performed across three independent grid regions per sample to account for spatial heterogeneity, and the resulting areal densities were averaged. These average values were subsequently used to construct a calibration curve relating SM density to analyte concentration.

### 2.3. Data Acquisition and Representation for Threshold–Based Counting

The input data consists of a time ordered fluorescence image sequence stored as a stack of TIFF frames. Each frame is a two-dimensional intensity image, and the full movie is represented as a three-dimensional tensor with spatial dimensions and a temporal dimension. Let *I* (*x*, *y*, *t*) denote the measured intensity at pixel location (*x*, *y*) in frame *t*, where *t*{1, …, *T*}. The analysis operates directly on the raw pixel intensities after constructing the full stack in memory.

### 2.4. Preprocessing and Noise Suppression

Fluorescence movies of SM blinking frequently exhibit high-frequency pixel-level noise together with low-frequency background structure. To improve the stability of the subsequent threshold-based detector, the pipeline applies median filtering to the image stack. Median filtering is a nonlinear denoising operation that suppresses impulsive noise while preserving localized structures more effectively than linear smoothing. In the implemented pipeline, the image stack was filtered using a three-dimensional median filter with kernel size (1, 3, 3), interpreted according to the internal array convention of the implementation. This corresponds to filtering within a small neighborhood while holding one dimension fixed and smoothing across the remaining two dimensions using the median operator. The filtered output is denoted as $I_{f}(x,y,t)$.

### 2.5. Threshold-Based Single Molecule Event Detection

SM emission events are operationally defined as temporally localized intensity transients that produce strong frame-to-frame changes compared with background fluctuations. The detector evaluates each pixel independently across time and searches for a three-frame temporal pattern (say, f_t_, f_t+1_, f_t+2_) consistent with a rapid change over consecutive interval.

**Figure 2:**
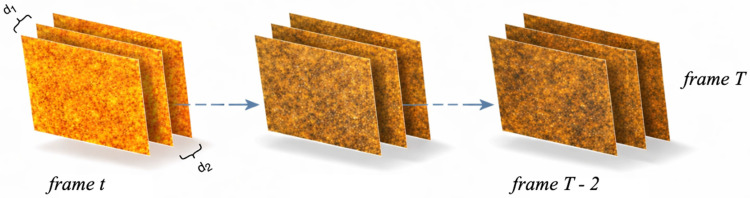
The progression of frames through the movie and visualization of intensity differences across the path.

For each pixel (*x*, *y*) and for each time index *t* from 1 to T − 2, three consecutive intensity values are extracted as depicted in Figure 2:

F_1_
*= I*_f_ (*x*, *y*, *t*), F_2_ = *I*_f_ (*x*, *y*, *t* + 1), F_3_ = *I*_f_ (*x*, *y*, *t* + 2)

Two absolute temporal differences are then computed:

d_1_ = ∣F_1_ − F_2_∣, d_2_ = ∣F_2_ − F_3_∣

A candidate detection is declared at pixel (*x*, *y*) if there exists at least one time index *t* such that:

d_1_ > τ and d_2_ > τ,

where τ is a user specified intensity threshold. Once the condition is satisfied for a pixel, the search over time terminates for that pixel, and the pixel is counted as detected. The final reported quantity for a given threshold is the total number of pixels in the field of view that satisfy the detection criterion at least once in the movie.

This definition explicitly targets strong temporal transitions that are unlikely under stationary background. It is also computationally efficient because it requires only local operations over three consecutive frames and permits early termination per pixel.

### 2.6. Implementation Details and Reproducibility

#### Computational Implementation and Workflow

**Figure 3:**
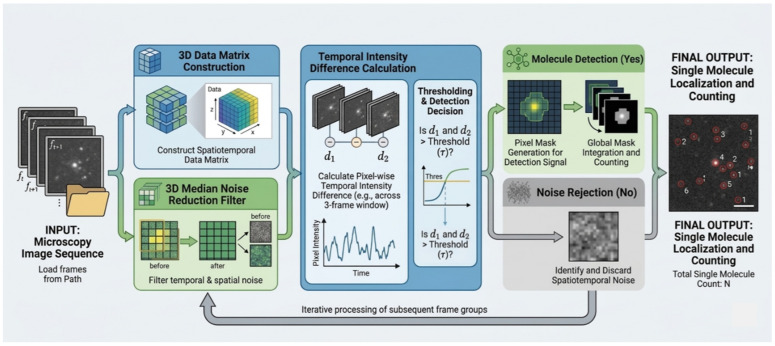
The methodology diagram for the threshold-based single molecule event detection.

The SM localization pipeline was implemented in Python 3.12.13 using Google Colab. NumPy version 2.0.2 was used for array operations, and SciPy version 1.16.3 was used for median filtering. The input microscopy sequence $\left(f_{t},f_{t+1},\ldots \right)$ was loaded from a TIFF file into a three-dimensional array with dimensions corresponding to x, y, and time as mentioned before. The pipeline then processed the data in four steps as illustrated in Figure 3.

1. Data Pre-processing and Denoising

The image frames were stacked into a 3D array so that spatial and temporal intensity changes could be analyzed within a single representation. As mentioned earlier, noise reduction was performed using scipy.ndimage.median_filter with kernel size (1, 3, 3), where the kernel dimensions correspond to the three axes of the stored image array. The filtered frames were saved as a separate TIFF sequence so that the exact preprocessed input used for detection could be retained.

2. Spatiotemporal Detection Logic

The detection step scanned the image over time using a three-frame window. For each position, the algorithm computed pixel wise intensity differences (d_1_, d_2_) across adjacent frames in order to isolate transient events. A candidate detection was recorded when the local signal exceeded a user defined threshold τ. Treating τ as a tunable parameter allowed the method to be evaluated under different signal to noise conditions.

3. Signal Selection

Pixels with intensity values above the threshold were retained and used to form a binary pixel mask representing detected signal locations. Pixels below the threshold were classified as noise and excluded from further analysis. This step reduced false positive detections in the final output.

4. Quantitative output

For a given threshold value, the pipeline returned the total number of validated detected pixels. As illustrated in Figure 3, threshold-positive pixels were first incorporated into binary signal masks and separated from rejected noise contributions, after which all retained detections were aggregated to produce the final automated SM count $\left(N\right)$. In the present study, these validated pixel-level transient detections constitute the operational counting units for threshold-based SM quantification.

### 2.7. Threshold-Based Analysis of Single Molecules: Comparison with Manual Counting

The detection of SMs in fluorescence microscopy is crucial for accurately quantifying molecular interactions, and automation of this process can significantly reduce manual labor. In this study, we compare manual counting with a threshold-based automated counting approach for detecting SMs across three fluorescent dyes, AF 488, RRX and AF 647. The threshold-based method employs changes in intensity criteria to identify SMs with minimal human intervention, leveraging the characteristic temporal signatures of SMs, such as rapid intensity changes, to distinguish them from background fluctuations.

## 3. Results

### 3.1. Outputs and Analysis Products

#### 3.1.1. Selection of Threshold

**Figure 4:**
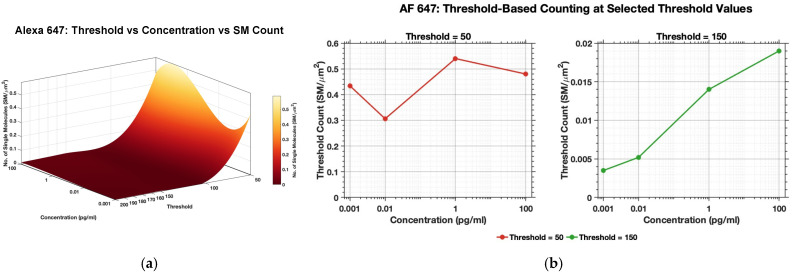
Threshold-screening analysis for Alexa Fluor 647. (**a**) Three-dimensional representation of threshold-based SM count as a function of concentration and threshold, used to identify threshold regions that preserve the expected concentration-dependent response while avoiding low-threshold overcounting and excessive high-threshold underdetection. (**b**) Two-dimensional concentration-response plots at representative threshold values of 50 and 150, illustrating the differing response behavior and the rationale for selecting threshold 150 for subsequent analysis.

**Figure 5:**
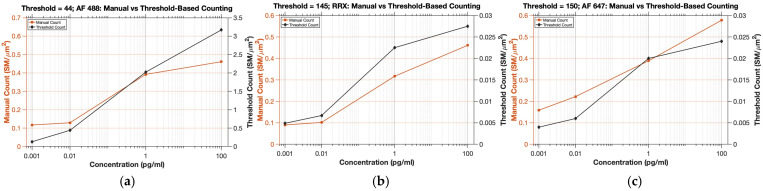
Post-selection validation of the threshold-based counting method against manual counting for AF 488 (**a**), RRX (**b**), and AF 647 (**c**) at the final selected thresholds. In each case, the threshold-based method preserves the same overall concentration-dependent trend observed in the manual counts, supporting the suitability of the selected thresholds for automated SM quantification.

Thresholds for AF 488, RRX and AF 647 were systematically optimized over a broad range of candidate values to identify operating points that maximized detection sensitivity while minimizing false positive counts arising from background fluctuations. For each dye, the selected threshold was chosen on the basis of its ability to reliably capture SM intensity transients across concentrations while preserving the expected concentration dependent increase in detected events. Thresholds below the optimal range tended to overestimate SM counts because of increased susceptibility to noise, whereas excessively high thresholds led to under detection of valid fluorescence events. The final thresholds were therefore selected as optimized values that provided consistent SM identification and maintained an increasing trend of the number of SMs and analyte concentration for each dye. Figure 4a further supports this threshold selection strategy by presenting the relationship between concentration, threshold, and threshold-based SM count in 3D surface form, whereas Figure 4b highlights the differing concentration response behaviors obtained at thresholds of 50 and 150, thereby justifying the selection of threshold 150 for subsequent analysis. The plots in Figure 5 demonstrate that both methods follow the same overall concentration dependent trend, while the threshold-based method yields a clearer and more strongly resolved increase in detected SM with increasing concentration. The 3D visualization further reinforces that the selected thresholds preserve the expected quantitative behavior of the assay and are suitable for automated SM counting.

#### 3.1.2. Comparison Between Manual and Threshold-Based Counting

**Figure 6:**
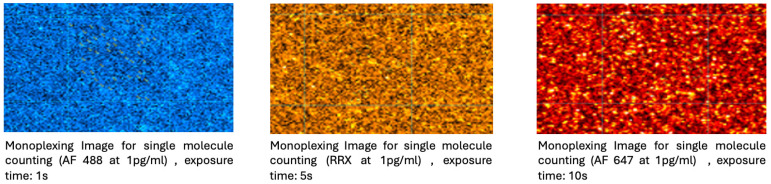
Manual counting for SMs obtained from ImageJ. The blinking pattern marks the presence of single molecules.

The comparison between manual and threshold-based counting methods for detecting SMs in fluorescence microscopy reveals several key insights into the strengths, limitations, and practical considerations of each approach. Both methods were evaluated using three fluorescent dyes, as mentioned earlier, with manual counting serving as the benchmark and threshold-based counting offering an automated alternative. The following section elaborates on the discrepancies observed between the two methods, the possible reasoning behind these discrepancies, and how each method contributes to the overall process of SM detection. The dataset used comprises sequential fluorescence microscopy frames arranged as a temporally ordered image stack, allowing the signal to be analyzed jointly across spatial coordinates and time. Because SM fluorescence experiments generate large datasets composed of high-resolution frames, for example 1920 × 1440 pixels, acquired over extended time sequences, manual counting and SM to background separation become inherently slow, labor intensive, and difficult to reproduce, as mentioned previously. Such analysis requires repeated frame-by-frame inspection over large image fields and depends on subjective identification of intensity peaks or transient blinking events, as illustrated in Figure 6, which shows manual counting of SMs in ImageJ based on blinking patterns. This increases susceptibility to analyst dependent variation and human error, particularly under low signal to noise conditions. By contrast, threshold-based counting applies predefined intensity criteria to classify fluorescence fluctuations as SM molecule events and can therefore evaluate large image stacks in a rapid, standardized, and reproducible manner. Although its performance depends on appropriate threshold selection and can be influenced by background noise, imaging conditions, and molecular dynamics, its computational scalability makes it a practical framework for rapid SM quantification.

To quantify the concentration-response relationship more explicitly, manual and threshold-based counts for each dye were fitted as linear functions of ${log}_{10}$-transformed concentration, where $x={log}_{10}(\mathrm{concentration}\;\mathrm{in}\;\mathrm{pg}/\mathrm{mL})$. As summarized in Table 1, manual counting showed strong linearity on the log-concentration scale, with $R^{2}$ values of 0.9272 for AF 488, 0.9729 for RRX, and 0.9958 for AF 647. Threshold-based counting likewise showed strong linearity, with corresponding $R^{2}$ values of 0.9888, 0.9465, and 0.9855. These results indicate that threshold-based counting reproduces the overall monotonic concentration-dependent trend observed by manual counting while providing a simpler and more scalable automated alternative for SM quantification.

#### 3.1.3. AF 488: Manual vs. Threshold-Based Counting

As shown in Figure 5a, both manual and threshold-based counting for AF 488 exhibited the same overall concentration dependent behavior, with the detected number of SMs increasing as the antibody concentration increased. The threshold for AF 488 was set to 44 following systematic evaluation of multiple candidate thresholds. The selection criterion was based on identifying a threshold that not only suppressed background contributions at low concentrations but also preserved the expected monotonic increase in SM counts with increasing antibody concentration. In particular, threshold 44 yielded the clearest concentration dependent separation across the tested samples, producing the most distinct differences between concentration levels while avoiding excessive background driven detections. This balance between concentration sensitivity and noise rejection supported its selection as the optimal operating threshold for AF 488.

Using this threshold, the threshold-based counts closely reproduced the trend observed by manual counting. Minor deviations were observed at lower concentrations, where manual identification of weak fluorescence transients was inherently more subjective because faint events were difficult to distinguish confidently from background fluctuations. By contrast, the threshold-based method applied a fixed and objective decision rule across all samples, improving consistency of detection. Although thresholding may still fail to capture events near the detection boundary, the selected threshold provided a robust compromise between sensitivity and specificity and, importantly, preserved the expected concentration response relationship of the assay.

#### 3.1.4. RRX: Manual vs. Threshold-Based Counting

Figure 5b shows that RRX channel likewise exhibited a clear increase in SM counts with increasing concentration under both manual and threshold-based analysis. A threshold of 145 was chosen because it produced the most stable concentration dependent progression while suppressing background driven detections. With this setting, the automated counts retained the same overall trend as the manual counts, although a systematic offset was observed, particularly at intermediate and higher concentrations. This difference likely reflects the increasing subjectivity of manual inspection when signals become weak, closely spaced, or partially crowded, conditions under which event boundaries are more difficult to judge visually. By contrast, thresholding applies to the same intensity criterion across all images, reducing observer dependent variation and yielding more consistent detection. These results indicate that the selected threshold captured the expected concentration response relationship while providing a more objective basis for SM quantification in the RRX channel.

#### 3.1.5. AF 647: Manual vs. Threshold-Based Counting

In the AF 647 channel, both methods again preserved the expected rise in detected SMs with increasing concentration as shown in Figure 5c, confirming that the concentration response trend was retained under threshold-based analysis. The final threshold was set to 150 using the same calibration framework applied to the other fluorophores, with selection based on preservation of a clear monotonic concentration dependent pattern together with suppression of background driven counts. Although the automated counts followed the same overall trend as the manual counts, they remained lower across the concentration range, and the difference became largest at the highest concentration. A likely contributor is the longer exposure used for AF 647 acquisition (10 s, compared with 1 s for AF 488 and 5 s for RRX). Longer exposure increases photon collection from true emitters, but it also increases integrated background fluorescence, detector noise, and nonspecific intensity fluctuations, thereby shifting the signal-to-background distribution toward higher cutoff requirements. The threshold ordering observed here is consistent with that expectation, although exposure time alone is unlikely to fully explain the differences, since fluorophore brightness, spectral characteristics, and channel dependent imaging conditions may also affect the final signal distribution. Under these conditions, a stricter threshold is required to limit false positive detection. The larger divergence at high concentration is also consistent with reduced reliability of manual inspection in denser or brighter regions, where adjacent emitters and background features may be visually overcounted. Thresholding, in contrast, enforces a fixed and reproducible detection rule and therefore yields a more conservative but more consistent estimate.

## 4. Discussion

While both manual and threshold-based methods provided generally consistent results, the key differences lie in their sensitivity to weak or overlapping SMs, as well as their susceptibility to human error. Manual counting, though accurate at higher concentrations, suffers from limitations in detecting low intensity SMs, especially at low concentrations or when the intensity of SMs is near the background noise level. Additionally, manual counting can be prone to subjective bias, particularly in dense regions of the image where closely spaced SMs may be mistaken for background noise or counted as a SM. A clear advantage of the automated analysis is the substantial reduction in processing time together with the expansion from partial field inspection to full movie analysis. For manual counting, each concentration was evaluated using only three selected grids out of approximately 690 total grids in a given frame. For manual counting, each concentration was evaluated using only three selected grids out of approximately 690 total grids in a given frame. At an experienced stage, counting a single grid required about 60 min, although this varied depending on frame quality, background intensity, and fluctuations in SM signal. This means that manual counting requires approximately 3 h per concentration per channel while covering less than 0.5% of the available spatial field. In contrast, the automated pipeline processed the entire movie of approximately 60 frames and analyzed the full image area rather than a small subset of selected grids. For a given threshold, one automated run required approximately 20 min to return the total number of detected SMs in the full movie. Thus, the automated approach reduced the analysis time from about 3 h to about 20 min per concentration per channel, corresponding to an approximately 9-fold improvement in efficiency, while also providing much broader spatial coverage. Notably, this estimate is conservative, as manual counting was performed on less than 0.5% of the available image area, whereas the automated pipeline processes the full field of view. In addition, manual counting was inherently labor intensive and susceptible to observer fatigue and expectation bias, particularly because the concentration was known during analysis and could influence judgment regarding whether the number of detected molecules should increase. The automated method avoided this subjectivity by applying the same detection logic consistently across all frames and concentrations. Threshold-based counting offers an objective, reproducible approach that removes much of human bias. However, its accuracy is highly dependent on the chosen threshold value. If the threshold is too low, the method may detect false positives, identifying background noise as SMs. Conversely, if the threshold is set too high, the method may fail to detect SMs, particularly at lower concentrations or when the fluorescence signal is weak. The method’s ability to maintain a consistent detection criterion across different experimental conditions makes it an attractive choice for large scale analysis, where human counting would be time prohibitive.

The present detector is most applicable to fluorescence movies in which true SM signals appear as temporally localized flicker-like transients rather than as persistent signals. Median filtering was adopted because it suppresses impulsive pixel-level noise while preserving localized structures more effectively than linear smoothing, whereas mean or Gaussian filtering may blur localized intensity changes and more adaptive approaches such as Wiener or bilateral filtering introduce additional assumptions or parameter sensitivity. At the same time, the method has practical limitations that follow directly from its reliance on flicker features. Its performance is reduced when true blinking cannot be clearly separated from other intensity fluctuations. Examples include persistent fluorescence, dense overlapping emitters, rapidly evolving structured background, motion or drift artifacts, photobleaching-related distortions, and detector-specific artifacts that mimic transient SM signals.

At the same time, it is important to distinguish the present framework from other simple automated strategies such as local-maximum peak finding, centroid localization, wavelet-based spot detection, and Gaussian fitting [49]. These approaches are effective when emitters appear as isolated point spread function (PSF)-like spots in individual frames with adequate photon counts and manageable background [50,51]. However, they rely on successful spatial spot identification and, in the case of Gaussian fitting, additional model assumptions and greater computational cost [51]. Their performance can also degrade under low signal-to-noise conditions, overlapping PSFs, or structured background [50,52]. In the present application, the primary objective is not subpixel localization of isolated emitters, but rapid and reproducible detection of temporally localized flicker-like transients in large fluorescence movies. The proposed threshold-based detector is therefore advantageous because it avoids explicit PSF fitting, local region-of-interest optimization, and emitter-by-emitter modeling, and instead uses a lightweight three-frame temporal-change rule that scales easily to large datasets. Within the counting definition adopted in this work, validated pixel-level transient detections constitute the operational units of threshold-based SM quantification. Thus, relative to other simple automated algorithms, the principal strength of the present method lies not in localization precision, but in operational simplicity, scalability, and direct sensitivity to transient blinking behavior.

## 5. Conclusions

The present study demonstrates that threshold-based SM detection provides a practical, reproducible, and analytically transparent alternative to manual counting in fluorescence microscopy. By replacing labor-intensive frame-by-frame inspection with a deterministic temporal decision rule, the proposed framework reduces analyst dependence, improves consistency, and enables efficient analysis of large spatiotemporal image datasets. The results further show that, despite its simplicity, the method preserves the overall concentration-dependent trends observed by manual analysis while offering clear advantages in speed, standardization, and scalability.

**Figure 7:**
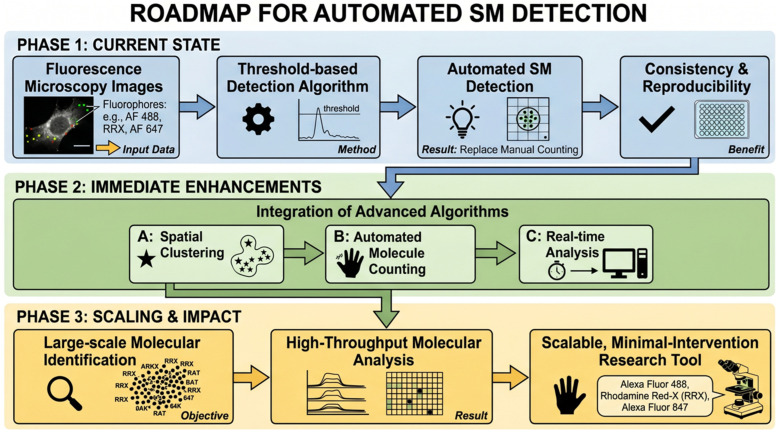
Visual roadmap for the operational transition from threshold-based automated single molecule detection to a fully integrated high-throughput pipeline.

Within the counting definition adopted in this work, validated pixel-level transient detections constitute the operational units of automated SM quantification. This formulation provides a clear and reproducible basis for automated counting without reliance on model fitting, training data, or complex localization workflows. At the same time, the framework also establishes a flexible platform for future methodological refinement. As summarized in Figure 7, subsequent development may incorporate spatial or spatiotemporal grouping of detections, automated molecule counting, and real-time analytical capability in order to further extend the range and applicability of the method.

Beyond these immediate developments, Phase 3 of the roadmap highlights the broader translational significance of the approach. With further refinement, the framework has the potential to support scalable high-throughput molecular identification, more efficient analysis of large fluorescence datasets, and reduced manual intervention in routine workflows. In this way, the present work establishes not only a useful automated alternative to manual SM detection, but also a strong foundation for future advances in molecular imaging, quantitative fluorescence analysis, and rapid automated diagnostic support.

## Figures and Tables

**Table 1:** Linear regression analysis of localization methods across experimental fluorescent labels.

Dye	Method	Fitted Equation	(R^2^)
AF 488	Manual	*y* = 0.0771*x* + 0.3326	0.9272
	Threshold	*y* = 0.6366*x* + 1.9175	0.9888
RRX	Manual	*y* = 0.0797*x* + 0.3023	0.9729
	Threshold	*y* = 0.0050*x* + 0.0191	0.9465
AF 647	Manual	*y* = 0.0845*x* + 0.4004	0.9958
	Threshold	*y* = 0.0033*x* + 0.0128	0.9855

## Data Availability

Data are contained within the article.
